# Association of coffee and tea consumption with the risk of lung cancer: a prospective cohort study from the UK Biobank

**DOI:** 10.3389/fnut.2025.1724041

**Published:** 2026-01-21

**Authors:** Shaozhong Zheng, Xiangying Suo, Chunlei Li, Shuo Lu, Jintao Han, Yacong Bo, Jing Guo

**Affiliations:** 1 Department of Thoracic Surgery, Puyang People's Hospital, Puyang, Henan, China; 2 Department of Clinical Nutrition, Zhengzhou University, Zhengzhou, Henan, China; 3 School of Sports Medicine, Wuhan Sports University, Wuhan, Hubei, China

**Keywords:** coffee intake, cohort study, lung cancer, tea intake, UK Biobank

## Abstract

**Background:**

The evidence of relationships between tea and coffee consumption with lung cancer risk remains inconsistent, with few prospective studies exploring dose-response relationships.

**Results:**

This prospective cohort study included 276,209 participants recruited from the UK Biobank (131,567 male and 144,642 female, mean age of 55.38 ± 8.01 years). Baseline coffee and tea intake was assessed via a touchscreen questionnaire. Hazard ratios (HRs) with 95% confidence intervals (CIs) were derived using Cox proportional hazards regression. Dose-response relationships were assessed via restricted cubic splines. During a median follow-up of 13.26 years, 3,821 participants developed lung cancer. The consumption of coffee and tea demonstrated non-linear associations with lung cancer risk (*P* for nonlinear < 0.001). Individuals consuming 0.5–1 cup (adjusted HR: 0.72, 95% CI: 0.64–0.81), or 2–3 cups (adjusted HR: 0.77, 95% CI: 0.69–0.86) of coffee daily had a lower risk of lung cancer relative to non-drinkers. Compared with non-tea drinkers, those who drank 0.5–1 cup (adjusted HR: 0.80, 95% CI: 0.70–0.93), 2–3 cups (adjusted HR: 0.67, 95% CI: 0.60–0.76) or ≥4 cups (adjusted HR: 0.86, 95% CI: 0.77–0.95) per day had a lower risk of lung cancer.

**Conclusions:**

This study demonstrated that moderate consumption of coffee and tea was associated with a lower risk of lung cancer. Further studies are needed to confirm these findings and elucidate underlying mechanisms.

## Introduction

As well as to being the second most prevalent type of cancer, lung cancer is the leading cause of cancer-related death. According to the most recent global cancer data, there were roughly 1.80 million cancer-related deaths and 2.20 million new cases globally in 2020 (1). Previous studies have suggested that most lung cancer patients are diagnosed at advanced stages, thereby having poor overall survival. Therefore, it is crucial for individuals to pinpoint modifiable risk factors, as these factors can serve as key targets to hinder the development of lung cancer.

In recent years, as awareness has been aroused on the role of diet in lung cancer, given that certain foods may precipitate or aggravate the morbidity and mortality of lung cancer (2). Coffee and tea, as beverages widely consumed in the UK and around the world, have been shown to have several effects on multiple human health outcomes (3–5). Specifically, coffee is not only a rich source of antioxidants and other bioactive compounds, but also contains additional nutrients beyond caffeine (6). Tea is rich in caffeine, catechin polyphenols, and flavonoids, and has been proven to possess anti-oxidative and anti-inflammatory effects (7). Previous studies have examined the association between coffee and tea consumption and lung cancer risk, but the findings remain controversial. Some studies have found that coffee consumption is associated with an increased risk of lung cancer (8–10), while tea consumption is associated with a reduced risk of lung cancer (9, 11). Meanwhile, other studies have not found a statistically significant association between coffee or tea intake and lung cancer risk (12–14). In addition, most of these studies were retrospective designs with relatively small sample sizes. To clarify these relationships, we prospectively examined the relationship between coffee and tea consumption and the risk of incident lung cancer based on a large national population-based cohort study from the UK Biobank.

## Materials and methods

### Study population

This study was based on the UK Biobank, a large prospective population-based cohort that recruited more than 500,000 participants aged 40–69 years in the UK, which has been described previously (15). In brief, a comprehensive baseline questionnaire survey and physical examination were conducted in 22 assessment centers in England, Wales, and Scotland between March 2006 and October 2010. A written informed consent form was obtained from all participants prior to recruitment. Ethical approval for their participation in the UK Biobank study was secured.

**Figure 1:**
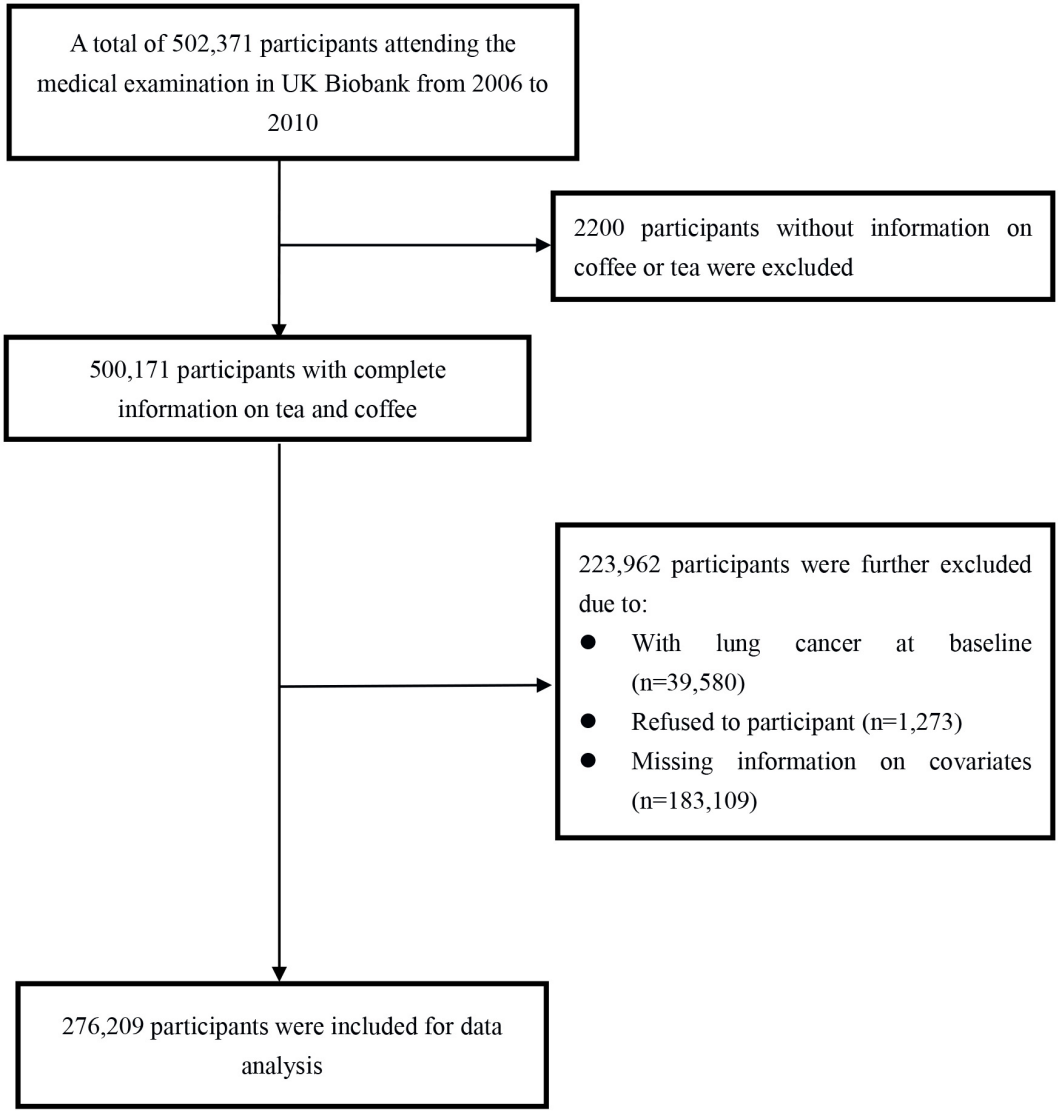
Flowchart of participant selection.

A participant selection process is shown in Figure 1. Briefly, a total of 502,371 participants aged 40–69 years were initially included from the UK Biobank (recruited from 2006 to 2010). We excluded 2,200 participants without information on coffee or tea intake, and an additional 223,962 participants due to a baseline diagnosis of lung cancer (*n* = 39,580), refusal to participate (*n* = 1,273), or missing information on covariates (*n* = 183,109). Finally, 276,209 participants were selected for the data analysis of the current study.

### Assessment of coffee and tea consumption

Coffee and tea intake was assessed at baseline using a touchscreen questionnaire. Participants were asked “How many cups of coffee do you drink each day? (Include decaffeinated coffee)” and “How many cups of tea do you drink each day? (Including black and green tea)”. Participants could select from the following options: “Less than one,” “Do not know,” “Prefer not to answer,” or “Specific number of cups”. If the answer was >10, participants would be asked to confirm their answers.

To investigate the association between coffee and tea consumption and incident lung cancer, we divided coffee and tea consumption into four categories (i.e., 0, 0.5 to 1, 2 to 3, and ≥4 cups/day). Participants who did not drink coffee (non-coffee drinkers) and those who did not drink tea (non-tea drinkers) were considered as the reference group respectively.

### Outcome ascertainment

The outcome for this study was incident lung cancer, which was determined by linking to cancer registries and death registries in England, Wales, and Scotland. Participants were followed up from recruitment until the date of lung cancer diagnosis, withdrawal from the study, the date of death, or last date of follow-up (October 2022 in England, August 2022 in Scotland, and May 2022 in Wales), whichever came first. Incident lung cancer was coded using the International Classification of Diseases (ICD) codes as C33 and C34.

### Assessment of covariates

The covariates in this study included demographic variables (sex, age, race, education level, and income) and potential confounders that may be associated with lung cancer, including body mass index (BMI), physical activity (PA), alcohol drinking, and smoking status. Race was divided into two groups: White and Other. Education level was divided into six categories: college or university degree, Advanced (A) levels/Advanced Subsidiary (AS) levels or equivalent, Ordinary (O) levels/General Certificate of Secondary Education (GCSE) or equivalent, Certificate of Secondary Education (CSE) or equivalent, National Vocational Qualification (NVQ) or Higher National Diploma (HND) or Higher National Certificate (HNC) or equivalent, and other professional qualifications. In this study, income was divided into five categories: less than £18,000, £18,000 to 30,999, £31,000 to 51,999, £52,000 to 100,000, and greater than £100,000. PA was classified into three categories: low-level PA, moderate-level PA, and high-level PA. To maximize the sample size, alcohol drinking and smoking status were divided into three categories respectively: current, former, and never. We additionally adjusted for baseline history of any cancer (excluding lung cancer), ascertained from hospital records, cancer registries, and self-report.

### Statistical analysis

The baseline characteristics are summarized as percentages for categorical variables, normal continuous variables by mean and standard deviation (SD), and non-normal continuous variables by median and interquartile range (IQR). Cox proportional hazards models were used to derive the hazard ratios (HRs) and 95% confidence intervals (CIs) of coffee and tea consumption separately with the risk of lung cancer. Three models were gradually adopted: Model 1: crude model without adjusting for any variables; Model 2: adjusted for age and sex; Model 3: further adjusted for race, physical activity, educational level, employment status, smoking, drinking, body mass index, townsend deprivation index, cardiovascular diseases (CVD), and cancer. To assess the dose-response relationship, a restricted cubic spline function was employed.

Cox regression analyses were conducted in subgroups defined by sex (male, female), age (< 65, ≥65), smoking status (never, ever), and BMI (< 25, ≥25) to evaluate potential modifying effects. Additionally, two sensitivity analyses were conducted to validate the robustness of these relationships: (1) Repeating the primary analyses after excluding participants diagnosed with lung cancer within 2 years after baseline; (2) Restricting the analysis to participants without cancer at baseline. All analyses were performed using R (version 4.0.0, R Foundation for Statistical Computing).

## Results

### Demographic characteristics

This study included a total of 276,209 participants. The mean age of the participants was 55.38 ± 8.01 years, of which 144,642 (52.4%) were female and 131,567 (47.6%) were male. Compared with participants who did not drink coffee or tea, those who did were more likely to be male, never alcohol drinkers, ever smokers, and to have a higher income level (Table 1).

**Table 1:** Demographic and lifestyle characteristics of 276,209 participants by coffee and tea intake category in the UK Biobank study.

**Characteristics**	**Overall**	**Coffee**				**Tea**			
		**0**	**0.5–1**	**2–3**	≥**4**	**0**	**0.5–1**	**2–3**	≥**4**
**Number**, ***n*** **(%)**	276,209	56,797 (20.6)	76,005 (27.5)	89,275 (32.3)	54,132 (19.6)	40,388 (14.6)	34,494 (12.5)	83,317 (30.2)	118,010 (42.7)
**Mean age (SD), y**	55.38 (8.01)	53.92 (7.95)	55.84 (8.02)	56.09 (7.98)	55.12 (7.87)	54.22 (8.05)	54.68 (8.20)	55.65 (8.07)	55.80 (7.84)
**Sex**, ***n*** **(%)**									
Female	144,642 (52.4)	32,904 (57.9)	41,644 (54.8)	45,500 (51.0)	24,594 (45.4)	21,959 (54.4)	17,070 (49.5)	43,273 (51.9)	62,340 (52.8)
Male	131,567 (47.6)	23,893 (42.1)	34,361 (45.2)	43,775 (49.0)	29,538 (54.6)	18,429 (45.6)	17,424 (50.5)	40,044 (48.1)	55,670 (47.2)
**Race and ethnicity**, ***n*** **(%)**									
White	156 (0.1)	28 (0.0)	35 (0.0)	63 (0.1)	30 (0.1)	19 (0.0)	28 (0.1)	49 (0.1)	60 (0.1)
Other	276,053 (99.9)	56,769 (100.0)	75,970 (100.0)	89,212 (99.9)	54,102 (99.9)	40,369 (100.0)	34,466 (99.9)	83,268 (99.9)	117,950 (99.9)
**Education**, ***n*** **(%)**									
College or University	118,672 (43.0)	21,351 (37.6)	33,645 (44.3)	41,199 (46.1)	22,477 (41.5)	16,250 (40.2)	16,926 (49.1)	37,437 (44.9)	48,059 (40.7)
A/AS levels	38,457 (13.9)	8,036 (14.1)	10,773 (14.2)	12,367 (13.9)	7,281 (13.5)	5,895 (14.6)	4,963 (14.4)	11,619 (13.9)	15,980 (13.5)
O levels/GCSEs	66,893 (24.2)	15,016 (26.4)	18,133 (23.9)	20,322 (22.8)	13,422 (24.8)	10,390 (25.7)	7,399 (21.5)	19,647 (23.6)	29,457 (25.0)
CSEs or equivalent	16,598 (6.0)	4,477 (7.9)	3,878 (5.1)	4,622 (5.2)	3,621 (6.7)	2,801 (6.9)	1,583 (4.6)	4,592 (5.5)	7,622 (6.5)
NVQ or HND or HNC	20,188 (7.3)	4,684 (8.2)	5,351 (7.0)	5,787 (6.5)	4,366 (8.1)	2,932 (7.3)	1,988 (5.8)	5,555 (6.7)	9,713 (8.2)
Other professional qualifications	15,401 (5.6)	3,233 (5.7)	4,225 (5.6)	4,978 (5.6)	2,965 (5.5)	2,120 (5.2)	1,635 (4.7)	4,467 (5.4)	7,179 (6.1)
**Median townsend deprivation index (IQR)**									
	−1.27 (3.07)	−1.58 (2.92)	−1.71 (2.86)	−1.63 (2.87)	−1.27 (3.07)	−1.35 (3.03)	−1.34 (3.04)	−1.61 (2.91)	−1.68 (2.85)
**Income**, ***n*** **(%)**									
< 18,000	42,266 (15.3)	10,176 (17.9)	12,186 (16.0)	12,216 (13.7)	7,688 (14.2)	6,435 (15.9)	4,619 (13.4)	11,669 (14.0)	19,543 (16.6)
18,000–30,999	66,090 (23.9)	13,872 (24.4)	18,501 (24.3)	21,224 (23.8)	12,493 (23.1)	9,534 (23.6)	7,470 (21.7)	19,734 (23.7)	29,352 (24.9)
31,000–51,999	79,871 (28.9)	16,457 (29.0)	21,517 (28.3)	26,062 (29.2)	15,835 (29.3)	11,761 (29.1)	9,889 (28.7)	24,073 (28.9)	34,148 (28.9)
52,000–100,000	68,778 (24.9)	13,242 (23.3)	18,447 (24.3)	22,788 (25.5)	14,301 (26.4)	9,982 (24.7)	9,341 (27.1)	21,461 (25.8)	27,994 (23.7)
>100,000	19,204 (7.0)	3,050 (5.4)	5,354 (7.0)	6,985 (7.8)	3,815 (7.0)	2,676 (6.6)	3,175 (9.2)	6,380 (7.7)	6,973 (5.9)
**Mean body mass index (SD), kg/m** ^2^									
	27.06 (4.58)	27.07 (4.82)	26.69 (4.49)	26.95 (4.41)	27.77 (4.65)	27.77 (5.05)	27.11 (4.67)	26.83 (4.45)	26.97 (4.45)
**Physical activity**, ***n*** **(%)**									
Low	51,373 (18.6)	11,100 (19.5)	13,362 (17.6)	15,593 (17.5)	11,318 (20.9)	8,329 (20.6)	6,737 (19.5)	14,975 (18.0)	21,332 (18.1)
Moderate	115,104 (41.7)	22,788 (40.1)	32,280 (42.5)	38,160 (42.7)	21,876 (40.4)	16,146 (40.0)	14,499 (42.0)	35,642 (42.8)	48,817 (41.4)
High	109,732 (39.7)	22,909 (40.3)	30,363 (39.9)	35,522 (39.8)	20,938 (38.7)	15,913 (39.4)	13,258 (38.4)	32,700 (39.2)	47,861 (40.6)
**Drinking**, ***n*** **(%)**									
Current	8,884 (3.2)	3,871 (6.8)	2,134 (2.8)	1,756 (2.0)	1,123 (2.1)	1,782 (4.4)	1,026 (3.0)	2,558 (3.1)	3,518 (3.0)
Ever	7,806 (2.8)	2,681 (4.7)	1,606 (2.1)	1,827 (2.0)	1,692 (3.1)	1,720 (4.3)	856 (2.5)	1,778 (2.1)	3,452 (2.9)
Never	259,519 (94.0)	50,245 (88.5)	72,265 (95.1)	85,692 (96.0)	51,317 (94.8)	36,886 (91.3)	32,612 (94.5)	78,981 (94.8)	111,040 (94.1)
**Smoke**, ***n*** **(%)**									
Current	157,913 (57.2)	35,395 (62.3)	45,554 (59.9)	50,565 (56.6)	26,399 (48.8)	22,444 (55.6)	19,253 (55.8)	48,446 (58.1)	67,770 (57.4)
Ever	92,761 (33.6)	16,931 (29.8)	25,245 (33.2)	31,193 (34.9)	19,392 (35.8)	13,233 (32.8)	11,696 (33.9)	28,406 (34.1)	39,426 (33.4)
Never	25,535 (9.2)	4,471 (7.9)	5,206 (6.8)	7,517 (8.4)	8,341 (15.4)	4,711 (11.7)	3,545 (10.3)	6,465 (7.8)	10,814 (9.2)
**Median tea intake (IQR), cups per day**									
	3.32 (2.76)	4.37 (3.19)	3.97 (2.57)	2.90 (2.27)	1.97 (2.55)	0.00 (0.00)	0.86 (0.22)	2.51 (0.50)	5.74 (2.40)
**Median coffee intake (IQR), cups per day**									
	2.04 (2.03)	0.00 (0.00)	0.87 (0.22)	2.40 (0.49)	5.25 (1.93)	3.36 (2.70)	2.80 (2.09)	2.02 (1.67)	1.39 (1.65)

### Coffee intake and tea intake with the risk of lung cancer

The association between coffee and tea consumption and risk of incident lung cancer is depicted in Table 2. After multivariable adjustment, both coffee and tea consumption were associated with a lower risk of incident lung cancer. Compared with non-coffee drinkers, daily consumption of 0.5–1 cup and 2–3 cups of coffee was associated with a 28% (HR 0.72, 95% CI, 0.64 to 0.81), and 23% (HR 0.77, 95% CI, 0.80 to 0.89) lower risk of lung cancer, respectively. However, participants who drank ≥4 cups coffee daily had no decreased risk of lung cancer (HR 1.03, 95% CI, 0.92 to 1.15). Compared with those who did not drink tea, those who drank 0.5–1, 2–3 and ≥4 cups of tea per day were associated with a 20% (HR 0.80, 95% CI, 0.70 to 0.93), 33% (HR 0.67, 95% CI, 0.60 to 0.76), and 14% (HR 0.86, 95% CI, 0.77 to 0.95) lower risk of lung cancer, respectively.

**Table 2:** HRs and 95% CIs for lung cancer by coffee and tea intake among the 276209 persons in the UK Biobank study.

**Characteristics**	**Coffee**				**Tea**			
	**0**	**0.5–1**	**2–3**	≥**4**	**0**	**0.5–1**	**2–3**	≥**4**
Number	56,797	76,005	89,275	54,132	40,388	34,494	83,317	118,010
Case, *N*	551	581	769	730	478	308	628	1,217
Model 1^a^	Ref	0.79 (0.70–0.89)	0.89 (0.80–0.99)	1.39 (1.24–1.55)	Ref	0.75 (0.65–0.87)	0.75 (0.65–0.87)	0.75 (0.65–0.87)
Model 2^b^	Ref	0.65 (0.58–0.73)	0.72 (0.64–0.80)	1.24 (1.11–1.38)	Ref	0.71 (0.61–0.82)	0.55 (0.49–0.62)	0.76 (0.68–0.85)
Model 3^c^	Ref	0.72 (0.64–0.81)	0.77 (0.69–0.86)	1.03 (0.92–1.15)	Ref	0.80 (0.70–0.93)	0.67 (0.60–0.76)	0.86 (0.77–0.95)

^a^Original model without adjusting for any variables. ^b^Adjusted for age and sex. ^c^Adjusted for age, sex, race, physical activity, educational level, employment status, smoking, drinking, body mass index, townsend deprivation index, cardiovascular, and cancer.

### Non-linear association

**Figure 2:**
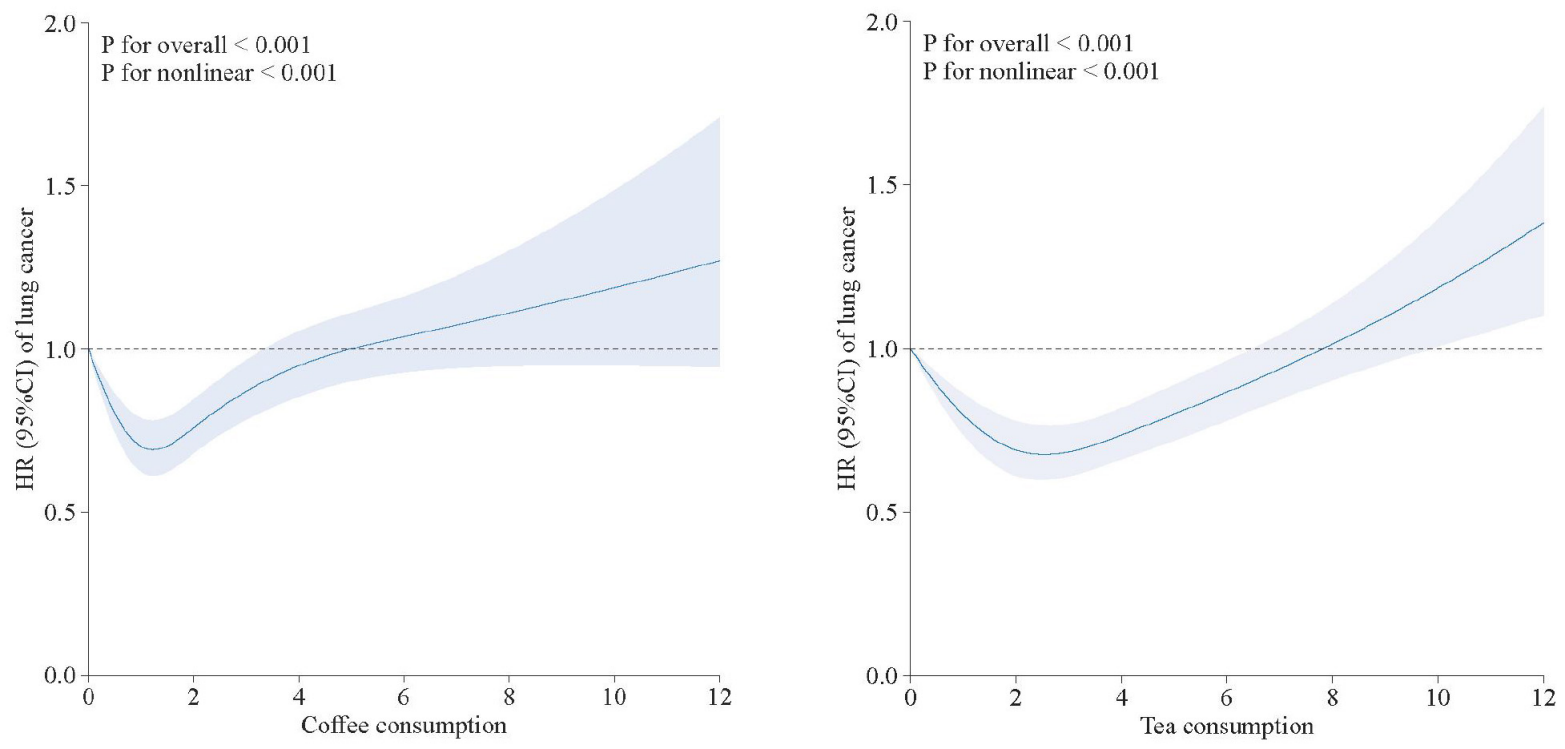
Concentration response between tea and coffee intake and the risk of lung cancer.

Restricted cubic splines were employed to assess the association between coffee and tea consumption and lung cancer risk. The multiple-adjusted models revealed a non-linear relationship between coffee and tea intake and lung cancer risk (*P* for non-linear < 0.001). Coffee intake of 1–2 cups/day or tea intake of 2–4 cups/day was linked with the lowest risk of incident lung cancer (Figure 2).

### Subgroup analysis and sensitivity analysis

The subgroup analysis results are presented in Table 3. The associations of coffee consumption with the risk of lung cancer statistically significantly differed by smoking status and BMI groups. However, the relationships of tea consumption with the risk of lung cancer statistically significantly differed between males and females and never and ever smokers. The sensitivity analyses also generally yielded similar results (Table 4).

**Table 3:** Stratified analysis HRs and 95% CIs for lung cancer by Coffee and Tea intake among 498,043 persons in the UK Biobank study.

**Characteristics**	**Coffee**					**Tea**				
	**0**	**0.5–1**	**2–3**	≥**4**	***P**_interaction_*	**0**	**0.5–1**	**2–3**	≥**4**	***P**_interaction_*
**Sex**										
Male	Ref	0.68 (0.58–0.80)	0.74 (0.64–0.87)	0.97 (0.83–1.13)	0.675	Ref	0.76 (0.62–0.93)	0.63 (0.53–0.74)	0.91 (0.79–1.06)	**< 0.001**
Female	Ref	0.77 (0.65–0.91)	0.80 (0.68–0.95)	1.13 (0.96–1.33)		Ref	0.86 (0.70–1.06)	0.86 (0.70–1.06)	0.86 (0.70–1.06)	
**Age**										
< 65	Ref	0.73 (0.64–0.84)	0.76 (0.67–0.87)	1.04 (0.91–1.19)	0.683	Ref	0.82 (0.69–0.97)	0.69 (0.60–0.80)	0.89 (0.78–1.01)	0.207
≥65	Ref	0.70 (0.56–0.87)	0.78 (0.64–0.96)	1.01 (0.81–1.25)		Ref	0.77 (0.59–1.00)	0.63 (0.51–0.79)	0.78 (0.64–0.95)	
**Smoker**										
Never	Ref	0.89 (0.70–1.13)	0.93 (0.73–1.17)	0.78 (0.58–1.04)	**< 0.001**	Ref	1.08 (0.77–1.52)	1.00 (0.75–1.32)	1.00 (0.77–1.31)	**0.036**
Ever	Ref	0.67 (0.58–0.77)	0.75 (0.66–0.85)	1.26 (1.11–1.42)		Ref	0.73 (0.62–0.86)	0.56 (0.49–0.64)	0.78 (0.70–0.88)	
**BMI**										
< 25	Ref	0.83 (0.68–1.01)	0.90 (0.74–1.09)	1.22 (1.00–1.48)	**0.008**	Ref	0.85 (0.67–1.07)	0.58 (0.48–0.71)	0.76 (0.64–0.91)	0.950
≥25	Ref	0.68 (0.59–0.78)	0.72 (0.63–0.82)	0.94 (0.82–1.08)		Ref	0.78 (0.65–0.94)	0.74 (0.64–0.86)	0.92 (0.81–1.05)	

Adjusted for age, sex, race, physical activity, educational level, employment status, smoking, drinking, body mass index, townsend deprivation index, cardiovascular, and cancer. Bold values indicates P < 0.05

**Table 4:** Sensitivity analysis HRs and 95% CIs for lung cancer by coffee and tea intake among the 276209 persons in the UK Biobank study.

**Characteristics**	**Coffee**				**Tea**			
	**0**	**0.5–1**	**2–3**	≥**4**	**0**	**0.5–1**	**2–3**	≥**4**
**Excluding lung cancer participants diagnosed within 2 years after baseline** ^*1^								
Number of participants	56,752	75,958	89,200	54,095	40,351	34,474	83,266	117,914
Number of case	506	534	694	693	441	288	577	1,121
Adjusted HR (95% CI)	Ref	0.72 (0.64–0.82)	0.76 (0.67–0.85)	1.06 (0.95–1.19)	Ref	0.82 (0.70–0.95)	0.67 (0.59–0.76)	0.86 (0.77–0.96)
**Excluding individuals with cancer at baseline** ^*2^								
Number of participants	3,707	5,541	6,192	3,322	2,592	2,148	5,685	8,337
Number of case	91	97	111	87	65	37	91	193
Adjusted HR (95% CI)	Ref	0.71 (0.53–0.96)	0.72 (0.54–0.95)	0.85 (0.63–1.15)	Ref	0.74 (0.49–1.11)	0.73 (0.53–1.00)	0.97 (0.73–1.29)

^*1^Adjusted for age, sex, race, physical activity, educational level, employment status, smoking, drinking, body mass index, townsend deprivation index, cardiovascular, and cancer.

^*2^Adjusted for age, sex, race, physical activity, educational level, employment status, smoking, drinking, body mass index, townsend deprivation index, and cardiovascular.

## Discussion

This large-scale cohort study, with a median follow-up duration of 13.26 years, is the first to identify a J-shaped relationship between coffee and tea consumption and lung cancer risk, with the lowest incidence associated with 1–2 cups/day or tea intake of 2–4 cups/day was linked with the lowest hazard ratio (HR) of incident lung cancer. To our knowledge, this represents the first population-based prospective cohort study in the UK.

Our research found that coffee consumption is associated with a lower risk of lung cancer. These findings are consistent with the results of a cohort study involving 24,528 Thai participants, which similarly demonstrated an association between coffee consumption and a lower risk of lung cancer (16). However, some previous studies revealed inconsistent findings, several studies from USA (12, 17), Korea (18), Norway (10), and Singapore Chinese (8), observed higher risk of lung cancer associated with coffee consumption, whereas two studies conducted in Japan found non-significant associations (13, 19). Similarly, our findings of the inverse relationships between tea consumption and lung cancer risk are consistent with several (8, 20) but inconsistent with some other studies. Among these studies inconsistent with our findings, some reported positive associations (14), while others revealed null connections (12, 19, 21). This inconsistency may be due to multiple factors, such as the variations in coffee or tea consumption among study populations, imprecision due to relatively small number of cases, differences in research methods, follow-up duration, or heterogeneity of study populations.

The inverse association between coffee and tea consumption and lung cancer risk may be explained by the complementary biological activities of their rich phytochemical profiles, which target fundamental cancer hallmarks like oxidative stress and chronic inflammation. Coffee exerts its effects through multiple compounds. Chlorogenic acids, for instance, are potent antioxidants that directly neutralize reactive oxygen and nitrogen species, thereby protecting pulmonary cell DNA from oxidative damage—a critical initiating event in carcinogenesis (22, 23). Other key components, including the diterpenes cafestol and kahweol, contribute by modulating metabolic pathways: they enhance cellular defense by inducing detoxifying enzymes and boosting antioxidant proteins, while simultaneously suppressing the activity of specific cytochrome P450 enzymes involved in procarcinogen activation (24). This coordinated shift helps protect lung tissue from DNA damage caused by inhaled carcinogens present in tobacco smoke and air pollution. Tea, particularly green tea, is distinguished by its high concentration of catechins, with epigallocatechin-3-gallate (EGCG) being the most bioactive. EGCG's chemopreventive properties are well-documented in preclinical studies. It potently suppresses the activation of key pro-inflammatory transcription factors, NF-κB and AP-1, leading to decreased production of cytokines (e.g., IL-6, TNF-α) and enzymes like cyclooxygenase-2, thereby attenuating the chronic inflammatory milieu that fosters tumor promotion and progression (25). Although the relative contributions of these pathways to lung cancer development require further clarification through integrated biomarker studies, the accumulated experimental and mechanistic data provide a robust, biologically plausible foundation for the protective associations observed in such population-based studies. This evidence underscores the potential role of dietary phytochemicals as components of broader strategies for cancer risk reduction.

This study is a large-scale prospective cohort study based on the UK Biobank, featuring a substantial sample size and extended follow-up period, providing epidemiological evidence for lung cancer prevention. Some limitations exist. Firstly, coffee and tea intake information was measured only once at baseline, rather than through multiple measurements during follow-up. Therefore, the risk of incident lung cancer associated with changes in coffee and tea intake could not be evaluated. Second, coffee and tea consumption was self-reported using a touch-screen questionnaire, which may lead to inaccurate responses, although the use of questionnaire to collect information is most popular in a large epidemiology study. Third, the majority of participants included in this study were of European ancestry; extension of research findings to other populations should be undertaken with caution. Fourth, these findings should be interpreted with caution when extended to other populations. Cohorts outside the UK may differ substantially in factors relevant to both beverage consumption and lung cancer risk. These include dietary habits (e.g., coffee roasting methods, types of tea, and consumption frequency), environmental exposures (such as air pollution levels and smoking prevalence), and genetic background. Such differences could alter the strength or even the direction of the observed associations. While the relative homogeneity of the UK Biobank population enhances internal validity, it may also limit generalizability to populations with markedly different cultural or environmental contexts. In addition, the use of registry-linked outcome data introduces several limitations, including potential misclassification or reporting delays, limited information on cancer stage, histological subtype, and treatment, and the possibility of incomplete ascertainment of very recent lung cancer diagnoses due to the periodic updating of registry linkages.

Despite these limitations, our study emphasizes that coffee and tea consumption are common and potentially modifiable lifestyle behaviors. Elucidating their associations with lung cancer risk may inform the development of population-based prevention strategies, particularly for individuals at elevated risk, such as smokers. Future research should seek to replicate these findings in more diverse populations, incorporate repeated assessments of beverage consumption to better capture long-term exposure patterns, and explore potential interactions with smoking status, genetic susceptibility, and environmental exposures. Such efforts will be essential for translating epidemiological evidence into targeted, evidence-based dietary recommendations for the prevention of lung cancer.

## Conclusion

The present study showed that compared to non-coffee drinkers, consuming ≤ 3 cups of coffee per day was associated with a lower risk of lung cancer, whereas consuming ≥4 cups of coffee per day was associated with a higher risk of lung cancer. Drinking tea was associated with a lower risk of lung cancer. Further research is needed to validate our findings and elucidate potential mechanisms.

In conclusion, we observed a significant non-linear association between coffee or tea consumption and incident lung cancer in a UK population, exhibiting a J-shaped pattern. However, given the weakened association observed regarding tea intake in the sensitivity analysis excluding baseline cancer patients, these findings should be interpreted with caution. Further studies are needed to validate these findings across different ethnic populations and elucidate the underlying biological mechanisms.

## Data Availability

The original contributions presented in the study are included in the article/supplementary material, further inquiries can be directed to the corresponding author.
